# Bone Augmentation in Rabbit Tibia Using Microfixed Cobalt-Chromium Membranes with Whole Blood and Platelet-Rich Plasma

**DOI:** 10.3390/ma8084843

**Published:** 2015-07-30

**Authors:** Oscar A. Decco, Víctor Beltrán, Jésica I. Zuchuat, Andrea C. Cura, María F. Lezcano, Wilfried Engelke

**Affiliations:** 1 Department of Bioingenieering, Faculty of Engineering, National University of Entre Ríos, Route 11 Km.10, Oro Verde (Paraná)-Entre Ríos 3100, Argentina; E-Mails: ceciliac@bioimplantes.com (A.C.C.); flezcano@bioimplantes.com.ar (M.F.L.); 2 CIMOFIR Research Centre, Faculty of Dentistry, University of La Frontera, Temuco 4781176, Chile; E-Mail: victor.beltran@ufrontera.cl; 3 Department of Maxillofacial Surgery, Georg-August-University Hospital, Robert Koch Str. 40, Göttingen D-37075, Germany; E-Mail: wengelke@med.uni-goettingen.de

**Keywords:** bone augmentation, cobalt-chromium membranes, microscrew, platelet-rich plasma

## Abstract

Background: Bone augmentation is a subject of intensive investigation in regenerative bone medicine and constitutes a clinical situation in which autogenous bone grafts or synthetic materials are used to aid new bone formation. Method: Based on a non-critical defect, Co-Cr barrier membranes were placed on six adult Fauve de Bourgogne rabbits, divided into two groups: whole blood and PRP. Three densitometric controls were performed during the experiment. The animals were euthanized at 30, 45, 60, and 110 days. The presence of newly formed bone was observed. Samples for histological studies were taken from the augmentation center. Results: External and internal bone tissue augmentation was observed in almost all cases. Significant differences between PRP- and whole blood–stimulated bone augmentation were not observed. At 60 days, bones with PRP presented higher angiogenesis, which may indicate more proliferation and cellular activity. Conclusion: PRP activates the bone regeneration process under optimized conditions by stimulation of osteoblast proliferation after six weeks, when a significant difference in cellular activity was observed. Membranes could stimulate bone augmentation at the site of placement and in the surrounding areas.

## 1. Introduction

Clinical management of many different types of disease, such as traumatic injuries, oncologic resection, congenital deformities, or progressive degenerative diseases (such as osteoarthritis, osteonecrosis, and periodontitis) is a challenge. The ideal treatment would allow for functional and structural reconstruction [1].

Regenerative medicine is an interdisciplinary branch of medicine that focuses on the repair, replacement, and regeneration of cells, tissues, or organs to restore a damaged function [2]. A major goal of regenerative medicine is the development of strategies that can be quickly applied to the clinic to solve problems that can benefit patients in the short and long term. The restoration of absent natural tissue by rebuilding damaged tissue organically and functionally is a topic of interest to clinicians, scientists, and bioengineers [3].

Bone substrate augmentation by regenerative techniques constitutes one of the most important research lines. Autogenous bone grafts and bone substitutes, biomaterials, membranes, and growth factors can be used alone or in combination to increase residual bone volume or regenerate absent bone, either on a predefined scaffold or in concert with a larger rehabilitation strategy.

Osteogenesis, osteoinduction, and osteoconduction can be used to optimize therapeutic approaches during regeneration and/or new bone formation [4].

In cases with critical bone defects that require repair and augmentation procedures, the use of autogenous bone grafts has been considered the gold standard, although the main disadvantage is that this requires removal of the patient’s own bone, increasing surgical complexity [5]. To overcome these drawbacks, allogeneic or xenogeneic grafts or fillers such as alloplastic bone substitutes were developed and are used in combination with barrier membranes [6,7,8,9]. Generally, traumatic bone lesions involve the breaking of blood vessels, which triggers the coagulation cascade and the formation of a blood clot. Clotting generates the accumulation of platelets at the site of the injury, and the consequent liberation of growth factors that aid tissue recovery and the generation of new bone. Previous studies by our research group [7] have shown that bone augmentation in rabbit tibia occurs in three months, and blood supply is promoted at the defect site by the clot.

Varkey M *et al.* (2004) [1] have studied the effect of the promotion of growth factors on bone formation. The use of platelet-rich plasma (PRP) in tissue engineering and regenerative medicine has the potential to aid the development of new biological products and techniques that improve the healing, regeneration, and formation of bone using the patient’s own tools. The capacity of bone regeneration or augmentation is limited and, with increased knowledge of bone growth factors, we could promote the differentiation, migration, and proliferation of the relevant cells.

The use of PRP is based on the release of multiple products of autologous plasma, such as growth factors (GFs) and reserve proteins. After the activation of platelets, these factors influence a range of processes that promote the recruitment, growth, and morphogenesis of bone cells.

Although the growth factors involved in the biology of PRP are poorly defined, in clinical practice, PRP has demonstrated benefits to patient treatment [10]. Several animal studies have demonstrated a beneficial effect of PRP in the repair of bone tissue. Some studies refer to bone formation around implants in the iliac crest of dogs [11], frontal bones of pigs [12], and in the jaws of mini-pigs [13].

Our goal is to create a favorable microclimate [14] at the site of injury. Osteoconductive and non-reabsorbable membranes favor osteoinductive mechanisms and cellular differentiation [7,15,16,17]. By placing a barrier membrane, the migration of non-desirable cells is prevented and desirable cells are allowed. These membranes constitute a medium to control the type of cells that migrate to the zone of injury, ensuring new bone formation and avoiding the proliferation of fibrous tissue [18].

The aim of the present study was to compare vertical bone augmentation from non-critical bone defects using microfixed Co-Cr membranes in samples with whole blood and PRP. Both membranes were placed in rabbit tibias, and our results were evaluated by macroscopic, histologic, and densitometric methods.

## 2. Results and Discussion

### 2.1. Results

**Figure 1:**
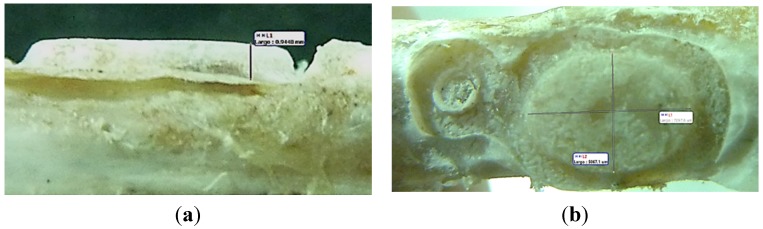
Macroscopic images of the augmented bone area in the rabbit tibia. (**a**) Lateral view used to measure bone augmentation height; (**b**) Top view used to measure width and length of the newly formed bone.

After the euthanize of each of the experimental animals, bone augmentation was observed (Figure 1) in almost all of them (with the exception of the tibia treated with PRP from first rabbit euthanized at 30 days, in which no internal augmentation was detected). We even sometimes observed bone augmentation over the Co-Cr membrane, in its surroundings, and at the micro-screw.

#### 2.1.1. Bone Measurement

After euthanize, close to the place of surgery, we first observed the existence or lack thereof of bone augmentation on the exterior surface of the membrane and in its surroundings. We determined three external measuring positions: P1 close to the micro-screw; P2 over the top of the membrane; and P3 close to the end of the membrane. We established four levels of description: not covered, starting of bone formation process in the borders of membrane, partially covered, and completely covered by bone (Table 1).

**Table 1:** Bone measurement.

Days	Whole Blood			PRP		
	P1	P2	P3	P1	P2	P3
30	Partially covered	Not covered	Not covered	Partially covered	Totally covered	Totally covered
45	Partially covered	Starting of bone formation process in the borders of membrane	Not covered	Not covered	Not covered	Not covered
60	Partially covered	Not covered	Partially covered	Starting of bone formation process in the borders of membrane	Starting of bone formation process in the borders of membrane	Not covered
110	Totally covered	Partially covered	Totally covered	Partially covered	Partially covered	Totally covered

Then, after detaching the membrane, the presence or absence of bone augmentation was evaluated. The height, width, and length of the newly formed bone was measured from macroscopic images to obtain the relationship between bone volume from inside of the membrane and its internal volume for each group (Table 2).

**Table 2:** Relationship between bone volume and the internal volume of the membrane.

Rabbit Number	Membrane Dimensions [mm] (width-long-depth)	Membrane Interior Volume [mm^3^]	Bone Dimensions [mm] (high-long-depth)	Bone Volume [mm^3^]	% (BV/MV × 100)
**Whole Blood**					
6	7.85 × 8.0 × 0.0	0.0	0.42 × 5.32 × 4.24	7.44	-
5	3.6 × 7.3 × 0.35	9.2	0.04 × 6.49 × 5.17	1.05	11.41
4	5.35 × 7.8 × 1.2	50.08	0.94 × 8.07 × 5.58	33.24	66.37
3	5.3 × 7.6 × 1.1	44.31	1.09 × 8.95 × 6.13	46.97	106.00
2	5.25 × 7.6 × 1.2	47.88	1.15 × 8.48 × 5.49	53.54	111.82
1	-	-	-	-	-
**PRP**					
6	5.85 × 10.0 × 1.75	102.37	0.0 × 5.89 × 3.97	-	-
5	3.95 × 5.75 × 0.55	12.49	0.92 × 0.75 × 0.44	0.24	1.92
4	4.5 × 5.85 × 0.95	25.01	0.52 × 4.53 × 3.07	5.68	22.71
3	5.5 × 7.8 × 1.1	47.19	0.97 × 7.95 × 5.61	33.98	72.01
2	5.35 × 7.55 × 1.15	46.45	1.02 × 7.8 × 5.7	45.35	97.63
1	4.2 × 5.9 × 0.75	18.59	0.57 × 7.45 × 5.4	22.93	123.35

#### 2.1.2. Densitometric Measurement

**Figure 2:**
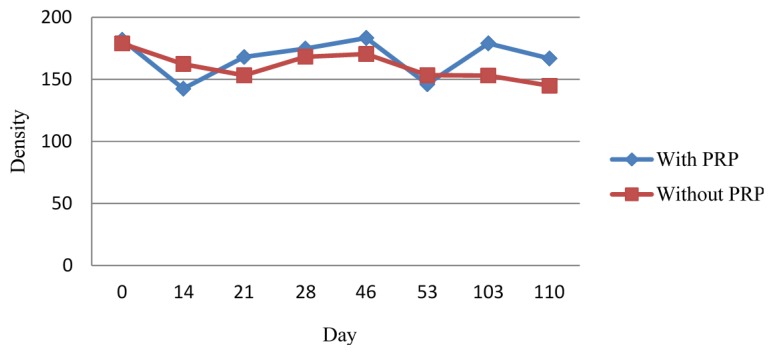
Average bone density at the center of the membrane.

Our aim was to quantify the density differences between samples with PRP and whole blood at different times (Figure 2). We used density as the dependent variable and the type of treatment (with and without PRP) as the independent variable.

A chi-square test was used to determine if any relationship between the treatments existed (with and without PRP), and the type of formed bone callus in each case (compact or compact and laminar bone). As p = 0.527 > 0.05, we observed no statically significant association between the treatment and type of bone callus obtained.

#### 2.1.3. Histological Examination

At 30 days post-surgery, after the euthanize of the first animal, high cellular activity in the extremity with whole blood was detected. We observed compact and laminar bone, with 200 osteoblastic cells on average. Preparations showed an excellent cellularity of osteocyte and osteoblast type and a homogeneous filling between cortical and interstitial. Areas surrounding the cells were found to have expression that would indicate non-mineralized collagen synthesis. In the extremity with PRP, only compact bone was detected, with 25 osteoblasts on average.

At 45, 60, and 110 days, higher cellular activity was observed in the tibia treated with PRP, with 480, 440, and 95 osteoblasts on average, respectively. In tibias treated with whole blood, we found 109, 224, and 10 osteoblasts on average.

The presence of osteons with good circumferential organization and laminar disposition compatible with cortical bone was observed in all tibias; additionally, Haversian formation was found, with expression of Volkmann and Havers canals of defined edges and concentric osteocytes. Laminar bone was observed in tibias with PRP from 45 days and only in tibias with whole blood at 30 and 60 days.

Differences in final vertical augmentation between both treatments were not observed and the histological appearance of the newly formed bone (cortical and laminar) was similar in both groups.

In the areas where bone was mature, cortical tissue with a good cellular concentration (basophilic coloration) was observed. Osteons and Haversian structures were identified with good morphologic, cellular, and matrix definition. In the immature areas, predominant osteoblasts were associated with the osteon formation. In the whole blood extremities, the bone synthesis process seemed to be stopped or decreased without osteoblastic fronts in the osseous surface; the bone presented a low osteosynthetic activity. Most of the mature bone tissue was found well-mineralized. In PRP samples, bone synthesis was presented very active from 45 to 60 days, decreasing in the latest period.

The presence of osteoclasts implicated in reabsorption and remodeling processes has not been detected, since we have not found any cells like this. Inflammatory signs or intolerance to PRP were not identified in any of the cases.

**Figure 3:**
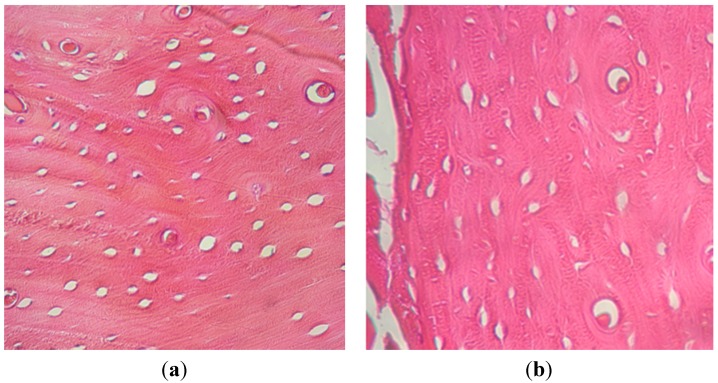
Histology at 60 days from tibias treated with: (**a**) whole blood; (**b**) PRP. (Original augmentation 40×; hematoxylin and eosin).

In the extremity treated with PRP at 60 days, an increased number of osteoblasts were observed compared to whole blood (Figure 3). We observed the proliferation of blood vessels and irrigation at the augmentation site in the PRP group from 60 days. However, in the opposite extremity, lower angiogenesis was observed. This vascularization had special characteristics, showing dilated vessels with thin walls and irregular contours sometimes associated with an osteoblastic front. This vascular dilatation was observed until the end of the study.

### 2.2. Discussion

PRP is used to augment bone regeneration volume due to its high concentration of platelets and consequent release of growth factors. In contrast with other osteoinductive agents such as bone morphogenetic proteins 2 and 7 (BMPs-2 and BMPs-7), autologous PRP causes no risk of allergies or tissue *versus* host reactions [19]. Growth factors found in PRP include platelet-derived growth factor (PDGF), transforming growth factor (TGFβ), vascular endothelial growth factor (VEGF), endothelial growth factor (EGF), insulin-like growth factor 1 (IGF-1), and platelet factor 4 (PF-4) [20,21]. *In vitro* experiments have proved that each of these factors produces different effects on bone formation [22]. The specific activities of PDGF, a factor found in high concentrations in platelets, include mitogenesis, angiogenesis, and macrophage activation. PDGF is not only responsible for recovery processes, but it is also used as a secondary source to continue bone repair and regeneration. TGFβ is produced in platelets and macrophages [23], acting as an autocrine and paracrine growth factor (activating adjacent cells, especially fibroblasts, bone marrow cells, and preosteoblasts, which can produce their own TGFβ). TGFβ can produce long-term recovery and bone regeneration, and even evolve into a bone remodeling factor over time [24]. The most important functions of TGFβ seem to be osteoblast chemotaxis and mitogenesis. TGFβ also inhibits osteoclast formation and bone resorption [25]. In an *in vitro* study on osteoblasts (“SaOS-2” type cells), Lavinia Casati *et al.* (2014) [26] showed that PRP controls the mechanisms responsible for cellular motion, increasing the chemokintics and chemotaxis, and also produces a temporary increase in the expression of the PDGF receptor.

The enrichment of PRP can influence the early stages of bone recovery. Given that platelets have an approximate lifespan of five-to-seven days, this influence decreases gradually [27,28] over the subsequent stages. Platelet action in the context of bone recovery works by two mechanisms: during the early stage, through the release of growth factors and cytokines; and later, by chemotaxis and macrophage activation [29].

Although the release of protein can last for one hour, the mean life of a growth factor is only a few minutes. After PRP is placed into the defect, a low oxygen cavity is formed, containing sufficient platelets, karyocytes, leukocytes, and collagen fibers adjacent to bone cells, osteoblasts, and mesenchymal stromal cells. The difference in oxygen levels between this cavity and the surrounding tissue could promote the migration of macrophages to the site [27,28].

Malhotra *et al.* (2013) [29] reported a decrease in the release of TGF-β, for which they suggested a possible decrease in platelet function when high centrifuging forces were used. A poor release of growth factors is attributable, generally, to an early activation and damage to the platelets during the purification process. To obtain higher concentrations of platelets, aggressive techniques can result in a lower quality of PRP. In an *in vitro* study, Choi *et al.* [30] found that high concentrations of PRP suppress the viability and proliferation of cells from alveolar bone.

In 2010, Miloro *et al.* [31] concluded that PRP does not provide any statistically significant benefit when was applied with osseous filling compared to PRP applied alone. However, in 2013, Daif *et al.* [32] studied the effect of PRP on bone regeneration in fractured jaws, concluding that the direct application of PRP over fracture lines can improve bone regeneration.

According to Anitua E *et al.* (2010) [33], the clinical use of PRP formulations is somewhat controversial because some studies conclude that the use of PRP may favor bone regeneration while others conclude that the use of PRP is irrelevant.

In a review by Roffi *et al.* (2013) [34], the authors state that there is no evidence to create clear conclusions about the role of PRP as an augmentation procedure. They establish that several aspects should be clarified; for example, which biomaterials can promote the effect of PRP, and which protocol for both for the production and application of the concentrate is ideal. Francesca Perut *et al.* (2013) [35] demonstrated that the biological activity of platelet concentrates varies according to the technique used to obtain it because the technique affects platelet content, leukocytes, and availability of growth factors. Meanwhile Massimo Del Fabbro *et al.* (2011) [36] assessed the results obtained from the use of autologous platelet concentrates in maxillary sinus augmentation. Half of the reviewed studies showed positive effects of PRP, and the other half did not find significant effects. The authors blamed heterogeneity of methods, biomaterials (osseous filling, for example), and the preparation of PRP, and did not claim a clear advantage in the use of PRP.

In addition to osteoinductive substances like PRP, several authors have studied the influence of membranes in bone augmentation procedures. Some authors have demonstrated positive effects in bone defect regeneration [4], and others have created a continuous debate as to whether a barrier membrane must be used to cover the augmented site [37]. The goals of membrane application are to prevent bone resorption, protect the graft from inhibitor factors and fibroblasts [38], maintain osteoconductive substances *in situ* [39,40], and allow the space to be gradually occupied by newly formed tissue [41].

Regarding the membranes manufactured for this study, bone formation is possible due to their engineering design and topography because these characteristics create a favorable cellular microenvironment to achieve bone augmentation. An important consideration is if the membrane architecture can influence osteoclast inhibition and osteoblast proliferation on its surface or in its surroundings. This remains an open question, and should be researched in future studies. Chen F-M *et al.* [14] observed that membranes prevented osteoblastic differentiation stimulated by the periosteum. A membrane’s surface topography alters osteoprotegerin (OPG) expression through adhesive MG63 cells [42], which causes signal transduction, transcription, regulation, proliferation, apoptosis, and cytoskeleton formation. Davies *et al.* [15] found that surface topography can affect osteoclastic number and activity, showing that the progenitor cells population is influenced by the membrane surface. The surface oxide could have a favorable effect on the extracellular matrix architecture and in the cellular activity next to the membrane. Placing occlusive, osteoconductive, and non-reabsorbable membranes makes bone formation easier [16,43], favoring the induction of augmentation and bone regeneration, and improving the results of techniques that use osseous substitutes [17].

Although several improvements in bone regeneration have been found, more tests are needed to determine if the use of PRP has statically significant benefits in clinical applications [31].

The purpose of our study was to stimulate vertical osseous growth in non-critical defects [44] through the combination of whole blood, PRP, and non-resorbable microfixed Cobalt-Chromium membranes. This microfixation was performed to prevent membrane displacement and stabilize the blood clot. In addition, the membrane acts as an extracellular matrix where cells can grow, and also contains growth factors [45].

In our macroscopic study, we observed a brown-colored bone augmentation at 60 days post-surgery in extremities with PRP; this coloration could be due to low osteoid mineralization [46] or high angiogenesis.

Bone volume was higher in extremities treated with whole blood, in all study stages.

In the first stage (0 to 30 days) of augmentation in the extremity treated with whole blood, we observed both laminar as compact bone and higher osteoblast numbers in the central augmentation area when compared to the extremity treated with PRP. In the PRP-treated limb, only compact bone with low osteogenic activity was observed.

Gerard *et al.* (2006) [47] concluded that PRP promotes the recovery process only during the early stage (first two months) when used with autografts in dog jaws. After this time, PRP does not have beneficial effects from the point of view of bone recovery, bone volume, or radiological density. In a later study, the same authors [48] concluded that PRP increases osteoblast and osteoclast numbers in the filling site only during the first month. From the second to sixth month, significant differences in the total osteoblast and osteoclast numbers between sites with and without PRP were not found. However, in our study, in the second stage (45 days), we found an increase in bone height and osteoblast numbers in the extremity treated with PRP. Microscopy studies suggested the presence of compact and laminar bone. Growth factors present in PRP may have provided the necessary conditions to favor angiogenesis, stimulating higher cellular activity. However, in the extremity treated with whole blood, a lower activity was observed, accompanied by a lower number of blood vessels and only compact bone.

At the third stage (60 days), the extremity treated with whole blood kept its growth speed, with a higher osteoblast number with respect to its counterpart from the previous stage. Meanwhile, in the opposite tibia, we found a small decrease in osteoblasts. In both legs, we detected the presence of laminar and compact bone.

Aghaloo *et al.* (2002) [49] reported the use of PRP in rabbit cranial bone defects; at the end of the study, the results did not show significant differences between defects treated with or without PRP. In our case, the bone with maximum height in both extremities was observed at the fourth experimental stage (110 days); this was determined by the membrane internal space. In tibias treated with whole blood, we observed fewer osteoblasts and compact bone with a greater mineralization. In the extremity treated with PRP, we observed higher cellular activity and the presence of laminar and compact bone. Thus, we observed a significant difference in relation to the average quantity of osteoblasts between tibias treated with PRP and treated with whole blood at the end of the experiment.

Different degrees of external bone formation were observed in most of the tibias, possibly due to the nature of the membrane and the surface treatment. With time, more bone tissue formed over the membrane, starting on the opposite extremes of the membrane.

The density study was performed using a Digora^®^ system. This tool gives the density at the point where we locate the cursor, added to the three densities of selected areas: minimum, mean, and maximum. In this case, the mean was used because it was more significant from the chosen section.

In locations where the membranes were placed, both legs showed a statistically significant median density augmentation, indicating that membrane placement introduces an additional factor in the density equation. If we correct for membrane placement using a constant value, we can infer that our results are representative because the density change between the different radiologic controls became independent using this constant.

By comparing the density of tibias with and without PRP at each time-point, and the difference between the beginning (post-surgery) and the end of the study, the only situation in which we observed significant differences in medians was in the control at 15 days. We observed that the group without PRP showed a statistically significant decrease in the median when compared to the group with PRP (p = 0.021 < 0.05). The other controls did not show significant differences.

A chi-square test was used to determine if any relationship between the treatments existed (with and without PRP), and the type of formed bone callus in each case (compact or compact and laminar bone). As p = 0.527 > 0.05, we observed no statically significant association between treatment and type of bone callus obtained. This is reasonable because the samples were taken at different post-surgery times.

From the density profile analysis over the augmentation in the vertical direction, we observed a higher density in the augmentation extremes close to the membrane, decreasing to the center. The same situation was found in the horizontal direction, where the density values decreased as we moved away from the bone surface. Chaves Netto *et al.* (2009) [50] performed a study in bone critical defects in dogs and, during the histological analysis of the samples, they found bone neoformation towards the center of the defect, characterizing a process of bone centripetal formation. This situation is similar to that found in our study because the zones with less density indicate recent bone formation, consistent with a tendency toward centripetal bone formation.

## 3. Experimental Section

Approval from the Superior Council of the National University of Entre Rios was obtained prior to the start of the study. Six adult Fauve de Bourgogne rabbits weighing between 2 and 2.8 kg were used as experimental animals. The animals were housed at 18–21° C with 50%–55% humidity. Each rabbit was housed in an individual cage. They were fed a standard commercial rabbit chow. Water and food were available *ad libitum*. The rabbit extremities were divided into two groups: group 1 (right extremity of each animal) had whole blood as the grafting material, group 2 (left extremity) had PRP. To provide space for the newly formed bone, approximately 1.5 mm deep Co-Cr membranes were used.

### 3.1. Surgical Procedure

General anesthesia was induced by an intra-muscular 5 mL dose of ketamine and 1 mL dose of xylazine; and local anesthesia was provided with 1.5 mL of carticaine L-adrenaline. Before the intervention, digital radiographs were taken of both tibias. The proximal metaphysis’ medial ridge of both the left and right tibia were shaved and disinfected with a povidone-iodine solution before the operation. This was followed by a full thickness skin incision and flap elevation exposing the tibial bone. For all tibias, a few microperforations were performed with a 1-mm diameter drill. For the right tibia, the membrane was placed and fixed with a vitallium microscrew. For the left tibia, PRP was placed between the membrane and the perforations. For the control animal, microperforations were performed on its right tibia, which was not covered by a membrane; only a Cr-Co membrane was placed on its left tibia. The flaps were repositioned and sutured with Vicryl 3.0 and Nylon 3.0 (Ethilon, Dilbeek, Belgium), and antibiotics were placed on the injury. The sutures were removed after two weeks. The animals were sacrificed at 30, 45, 60, and 110 days after the intervention with an overdose of sodium pentobarbital IV (Dolethals; Vetoquinol, Lure, Saint Anne, Alderney, France).

### 3.2. PRP Preparation Technique

Blood from each rabbit was centrifuged in a Zelian centrifugal TYFON II for 8 min at room temperature for separation of cellular components. The plasma layer was extracted and placed in a sterile test tube and centrifuged again under same conditions to separate platelet rich plasma (PRP) from platelet poor plasma (PPP). The PPP corresponds to the upper portion, while the PRP stays at the base of the tube. PRP was placed in a sterile tube with 0.025 M calcium chloride for clot formation.

### 3.3. Analysis

Macroscopic assessment of the animals was performed once a week.

For densitometric analysis, the Digora^®^ system was used to obtain density values by digital radiology. This information was then processed by sign and chi-square tests using SPSS^®^ statistical software. Each image was measured according to the gray levels expressed through the “density measurement” function in delimited areas. In all cases, the selected area was the bone surrounding the micro-screw and membrane, with a homogenous selection of 10 × 10 pixels (0.7 mm^2^). Five positions at different distances from these elements were used. Densitometric studies were performed before and after the surgery (the same day), at 15 days post-surgery, and after euthanize on each specimen. 

Post-mortem, after detaching the membranes, the augmented tissue was measured using a stereo microscope and Motic Images Plus^®^ software. The samples were fixed in a 10% formaldehyde solution for 24 h for further analysis. Samples for the histological study were taken with a trephine of 1 mm diameter from every augmentation center; in case of no augmentation, a sample was taken from the central region that was covered by the membrane. Tissue samples containing the newly formed bone were removed for routine laboratory processing for decalcified sections, with inclusion in paraffin. After inclusion, the blocks were set on their slides and stained with hematoxylin and eosin.

Results are expressed as the mean number of osteoblasts counted in 10 microscope fields uniformly obtained from slides at power magnification (×40).

## 4. Conclusions

We observed that both procedures stimulated bone augmentation at the application site by the end of the experiment. We did not observe significant differences between treatments. However, at 60 days, we observed that extremities with PRP showed higher angiogenesis, which might indicate higher proliferation and cellular activity. Corresponding with results from our histological study, we observed twice as many osteoblasts in the tibia treated with PRP compared to whole blood at the same period of time.

We conclude that PRP may be able to activate the bone regeneration process in optimized conditions by stimulation of osteoblast proliferation at the sixth week, where a significant difference in cellular activity was observed.

In the control animal where only a Co-Cr membrane was placed, the presence of cellular activity was observed with cortical and laminar bone formation, although with a lower velocity as in the previous cases. We infer that the membrane stimulates bone augmentation at the placing site and in the surrounding zones. This characteristic could be used as starting point in future studies to investigate the inductor capacity of such a surface. From a cell and molecular biology perspective, it would be interesting to determine the influence of different growth factors in bone augmentation.
